# Microplasma Controlled Nanogold Sensor for SERS of Aliphatic and Aromatic Explosives with PCA-KNN Recognition

**DOI:** 10.1021/acssensors.4c02651

**Published:** 2024-12-24

**Authors:** Jaka Olenik, Vasyl Shvalya, Martina Modic, Damjan Vengust, Uroš Cvelbar, James L. Walsh

**Affiliations:** † York Plasma Institute, School of Physics, Engineering and Technology, University of York, York YO10 5DD, U.K.; ‡ Department for Gaseous Electronics F6, Jozef Stefan Institute, 1000 Ljubljana, Slovenia

**Keywords:** gold nanoparticles, plasma synthesis, SERS, explosives, machine learning

## Abstract

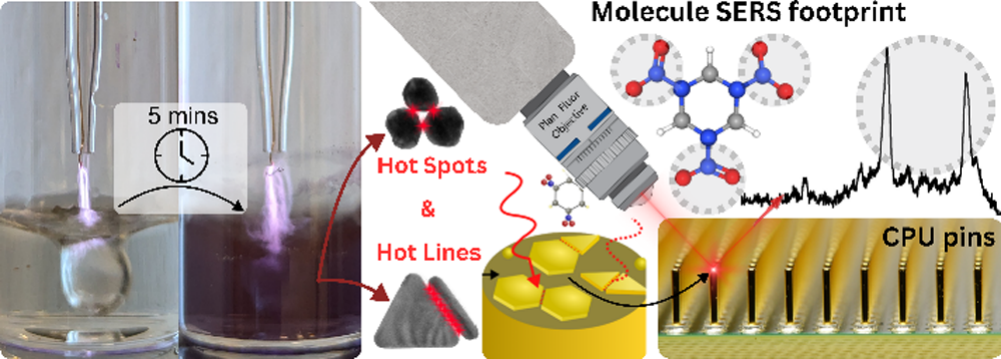

Nanogold is an emerging material for enhancing surface-enhanced Raman scattering (SERS), which enables the detection of hazardous analytes at trace levels. This study presents a simple, single-step plasma synthesis method to control the size and yield of Au nanoparticles by using plasma-liquid redox chemistry. The pin-based argon plasma reduces the Au^3+^ precursor in under 5 min, synthesizing Au spherical particles ranging from ∼20 nm at 0.025 mM to ∼90 nm at 1.0 mM, in addition to plate-like particles occurring at concentrations of 0.25–1.0 mM. The enhanced SERS responses correlated with the UV–vis absorption and reflectance profiles, which can be attributed to synergistic plasmonic hotspots created by the sphere–sphere, plate-sphere, and plate–plate nanogold interactions. This nanogold mixture, combined with gold-plated CPU grid pin arrays, facilitated the detection of trace explosives, including aromatic (TNT, TNB, and TNP) and aliphatic (RDX, PETN, and HMX) compounds. We demonstrate that stabler aliphatic analytes, associated with lower vapor pressure (10^–8^–10^–11^ atm), exhibit smaller signal fluctuations (RSD ∼ 6–10%) compared to their more volatile (10^–5^ atm) aromatic (RSD ∼ 12–17%) counterparts at similar analyte concentrations. The calculated limit of detection (LoD) was found to be ∼2–6 nM and ∼600–900 pM for aromatic and aliphatic explosives, respectively. Finally, we show that the poorer performance of aromatic explosives under the same sensing conditions affects SERS-PCA separation, which can then be improved either by a machine learning approach (PCA with k-NN classification) or by consideration of a specific NO_2_ symmetric stretching fingerprint range.

Spectroscopic methods such as surface-enhanced fluorescence (SEF), surface-enhanced infrared absorption (SEIRA), and especially surface-enhanced Raman scattering (SERS) have proven disruptive in the field of analytical chemistry, enabling the swift detection of hazardous analytes and, more importantly, at trace levels, approaching the single-molecule detection regime. Such developments have become possible through advances in the development of plasmonic materials with different nanoscale features, either in the form of nanostructured thin films or nanoparticles, with gold being a popular choice when the stability of sensing performance over time is of primary analytical importance.^1−5^ For military and security applications, these criteria, together with robustness under extreme conditions and minimal data acquisition time, are of particular importance for the development of sensors that can be deployed under real-world conditions. With a constantly increasing number of synthetic explosives, armed conflicts, and their potential escalation, there is a growing need to develop methods that accurately detect the presence of explosives and their toxic products at trace levels in the environment.^6−9^

To date, the presence of explosive vapors, traces of substances, and toxic chemical warfare agents are typically quantified using capillary electrophoresis, ion mobility spectroscopy, and gas chromatography-based methods.^10−14^ However, SERS approaches have the potential to outperform these classical methods in many critical parameters, such as detection time, portability, and sensitivity.^15,16^ Piorek et al. demonstrated rapid and label-free identification of 2,4-DNT in the vapor phase using real-time Raman fingerprinting, achieving a parts per billion detection limit using Au NPs with an average size of 35 nm.^17^ In another study, faceted Au octahedra (∼40 nm) separated by voids of less than 10 nm served as electromagnetic hotspots yielding a profound field enhancement enabling the analysis of nitroaromatic TNT explosives at low concentrations (10^–9^ M).^18^ The SERS performance of functionalized nanospheres and triangular nanoprisms (both made of Au with a size of ∼30 and ∼100 nm, respectively) was compared by detecting the presence of DNT in aqueous media by Xu et al. In their study, the resulting signal enhancement of the plasmonic nanoprisms (1.05 × 10^8^) was about an order of magnitude higher than that of spherical NPs (2.13 × 10^7^). This 10-fold increase was attributed to the intense EM field confinement at the tips of the NPs.^19^

Although Au nanospheres show adequate SERS performance, they exhibit limited plasmonic peak tailoriability and are inferior in terms of analytical performance compared with more complex NPs with planar shapes (triangles and hexagons). Such particles can provide hotline electric field confinement when tightly arranged in a side-by-side conformation. Many reports describe the synthesis of spherical particles;^20−22^ however, this does not apply to planar Au shapes, for which only complex multistep and time-consuming procedures with external reagents and stabilizers have been thoroughly described.^23−25^

Plasma-based synthesis tools have recently been introduced to enhance nanogold design in terms of production time and size control.^26^ For example, an inductively coupled radiofrequency (RF) plasma at 13.56 MHz operating in low-pressure argon (20–200 Torr) was used by Izadi *et.al.* to produce monodisperse AuNPs with a size of less than 10 nm by degrading a gold wire.^27^ Chantaramethakul et al. utilized solution plasma sputtering (20 kHz) with two Au wire electrodes (in a Pyrex glass reactor) as the Au source. They achieved control of the particle size in the range of 5–20 nm but demonstrated limited control over particle shape, which was predominantly spherical.^28^ Distorted spherical gold nanoparticle agglomerates <20 nm were obtained using an atmospheric pressure DBD plasma torch, employing a nebulized salt solution with argon serving both as a carrier and discharge-forming gas.^29^ Maguire et al. reported on the use of RF plasma for the nebulizer-assisted in-flight synthesis of small Au nanoparticles (∼4 nm) using He as the plasma-forming gas and Ne as the microdroplet precursor carrier.^30^ In the reports of Li et al., Saito et al., and Heida et al. a direct plasma discharge was used between the cathode and the solution surface, revealing the first signs of flat Au shape formation; while such results are intriguing, further efforts are required to support the development of improved SERS sensors.^31−33^

In this report, we demonstrate that planar shapes of nanogold can be synthesized with improved yields by exploiting plasma-liquid redox chemistry. Using a single-step atmospheric pressure plasma fabrication approach with varying Au^3+^ precursor concentration within an order of magnitude 0.025–1 mM, the plate/sphere geometry ratio was improved significantly. To demonstrate the enhanced plasmonic performance of the material, an experimental case study employing SERS was performed to rapidly detect and distinguish between multiple aliphatic and aromatic trace-level explosives. To enable rapid detection of multiple analytes, we exploited a legacy, pin-based, CPU from a desktop computer as a substrate, providing an abundance of well-isolated gold-coated surfaces on which to deposit Au nanoparticles. The well-defined pattern of microchip pins, in conjunction with plasmonic scatterers, showed exceptional performance in the acquisition of SERS data for a range of explosives, including TNT, TNP, TNB, HMX, RDX, and PETN. To distinguish between explosives, the acquired spectra were dimensionally reduced utilizing principal component analysis, and the resulting components were used to train a machine learning model based on the k-nearest neighbors algorithm.

## Experimental Section

### Plasma Characterization and Nanoparticle Preparation

**Figure 1:**
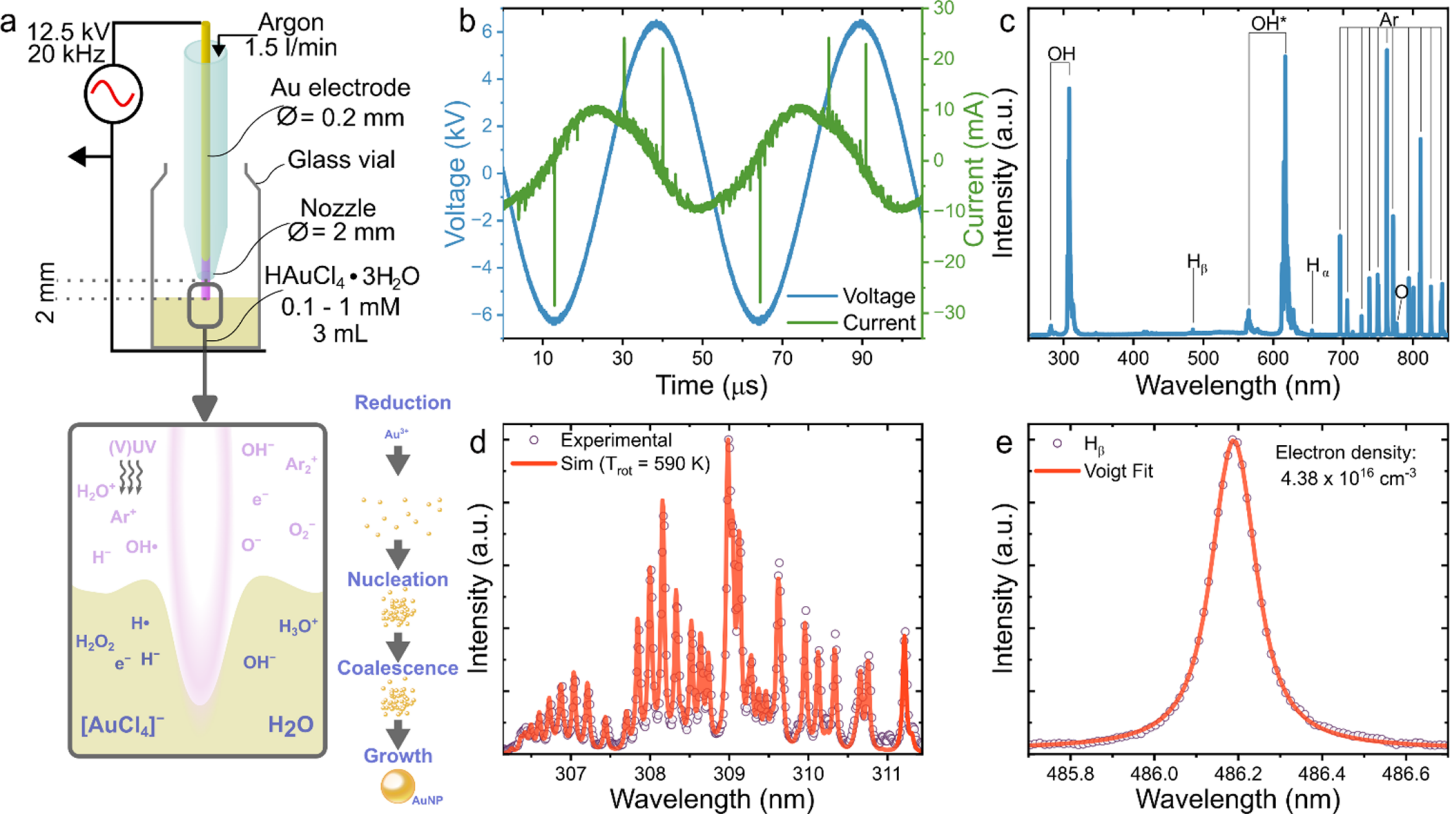
(a) Schematic showing the cold atmospheric pressure plasma (CAP) setup with a diagram highlighting the plasma-liquid interface and proposed particle formation process; (b) voltage and current waveforms during plasma generation; (c) emission spectrum of CAP at the liquid interface; (d) simulated (*T*_rot_ ≈ 590 K) and experimental spectra of excited OH; and (e) measured H_β_ emission line and Voigt fit for *n*_e_ calculation.

The plasma-generating electrode was composed of a single gold wire electrode (200 μm diameter) housed within a t-shaped glass tube (6 mm diameter), one end of which was formed into a nozzle with a 2 mm exit diameter. The free end of the glass tube was connected to an argon gas supply, and the entire electrode unit was sealed to ensure gas only exited through the nozzle. The exit nozzle was oriented in a vertical downward direction and positioned approximately 5 mm above the surface of the aqueous precursor used in synthesis experiments. In all experiments, the argon flow at 1.5 L/min was initiated 60 s prior to plasma generation to ensure air from within the vial was removed. The plasma system used to generate the necessary high voltages to strike and sustain a discharge consisted of a homemade switched-mode power source and high voltage transformer, capable of operating at 20 kHz and generating voltages exceeding 12 kV in amplitude. The output of the transformer was connected to the Au wire electrode, while the glass vial containing the aqueous precursor was positioned on a grounded metallic stage, acting as a counter electrode, as shown in Figure 1a. During the plasma-assisted synthesis, the voltage and current waveforms were monitored on an oscilloscope (MSO5000, Rigol) using a high voltage probe (P6015A, Tektronix) and a current probe (model 2877, Pearson Electronics). Probes were positioned between the high-voltage source and the gold wire electrode. The tetrachloroauric(III) acid trihydrate HAuCl_4_·3H_2_O, purchased from Sigma-Aldrich (CAS Number: 16961–25–4), was dissolved at room temperature in Milli-Q water to obtain a concentration set of 0.025, 0.050, 0.075, 0.1, 0.25, 0.5, 0.75, 1.0, and 2.5 mM. Solutions were sonicated for 10 min and centrifuged for 10 min at 14,000 rpm (20817 RCF) to remove precipitations before plasma treatment. For all tests, a total treatment time of 5 min was adopted. Immediately after each treatment, the temperature of the solution was measured using a K-type thermocouple and found to be between 36 and 37 °C, indicating minimal thermal effects. Optical emission spectroscopy (OES) measurements of the plasma were obtained under identical conditions to those used in the NP synthesis experiments, with the exception of a quartz cuvette being used rather than a glass vial, thus facilitating the transmission of emissions at wavelengths <350 nm. Emissions were captured through the side window of the cuvette using an optical fiber (M92L02, Thorlabs) positioned approximately at the plasma-liquid interface and connected to a spectrograph (Shamrock SR500i-D2-R, Andor) equipped with an iCCD camera (iStar 334T CCD, Andor). A broad spectral scan in the range of 250–850 nm with a 300 lines/mm grating was used to ascertain excited species. Higher resolution scans with a 2400 lines/mm grating were made of the OH(A–X) emission spectrum in the range 306–312 nm and the hydrogen Beta line (486.13 nm) which was used to calculate the rotational temperature and electron density, respectively.

### Characterization of the Nanoparticles

UV–vis absorbance measurements were conducted using a microplate reader (Spark 10 M, Tecan, Männedorf, CH), analyzing 0.2 mL of each analyte across the spectral range of 200–1000 nm. Total diffuse reflectance spectra of aqueous precursor solution before and after plasma treatment were obtained with a double beam UV–vis spectrophotometer (model 1050, Perkin Lambda) equipped with a 150 mm diameter InGaAs integrating sphere module. Spectra in the range of 500 and 700 nm were scanned with a resolution of 0.2 nm. The size and shape of plasma synthesized gold nanoparticles were investigated with scanning electron microscopy (SEM, Prisma E, Thermo Fischer Scientific), equipped with ETD and CBS detectors (EDS, model Inca 400, Oxford Instruments). SEM micrographs were taken at 10 kV. The detailed morphology was later revealed with transmission electron microscopy (TEM, JEM-2100, Jeol), operating at 200 keV. Alongside SEM and TEM, a multiangle dynamic light scattering system (MADLS, Zetasizer Ultra, Malvern Panalytical) was used to ascertain the particle size and their concentration in aqueous solution. Measurements were taken of 1 mL of nanocolloids at room temperature immediately after the synthesis. COMSOL Multiphysics was used to model the electric field distribution for different conformations of nanoparticles. X-ray photoelectron spectroscopy was used for the reduction efficiency of the plasma treatment. The spectrometer (PHI-TFA XPS, Physical Electronics) was equipped with an Al- monochromatic X-ray source with a pass energy of 1486.6 eV and active surface charge neutralization. A highly concentrated sample of 1.0 mM before and after treatment was drip deposited onto a clean Si wafer, dried, and then placed in the chamber.

### SERS Measurements and Classification Algorithm

Surface Enhanced Raman Spectroscopy was performed with a confocal Raman spectrometer (NTEGRA Spectra II, NT-MDT Spectrum Instruments) employing a He–Ne laser operating at a wavelength of 633 nm with a slit of 100 μm. An RMS20X - 20× Olympus Plan Achromat objective, 0.4 NA, 1.2 mm WD was used for the investigation. A crystal violet marker was prepared in the concentration range 10^–5^–10^–9^ M for limit of detection testing. Other marker molecules used in the study included Rhodamine R6G, Alcian Blue, and Xylenol Orange, diluted in water at a concentration of 10^–6^ M. Prior to SERS analysis, a laboratory centrifuge (10,000 rpm or 10621 RCF for 5 min) was used to concentrate particles and the remnant water removed. Then Raman molecules were added to nanogold sediments and pipetted onto CPU pins (Intel i386), dried at ambient conditions, then measured. In a similar manner the spectra for explosives were recorded. For SERS and machine learning classification, 3 aliphatic (RDX, HMX, PETN) and 3 aromatic analytes (TNT, TNP, TNB) were purchased from AccuStandard at a concentration of 1 mg/mL and mixed with nanogold colloids after centrifuging. Dilution series were prepared in water. Complementary UV–vis measurements of the TNB explosive were conducted using a microplate reader (Spark 10 M, Tecan, Männedorf, CH), analyzing 0.3 mL of each analyte across the spectral range of 200–450 nm. Raman data preprocessing, statistical analysis and machine learning was done in Python programming language using its openly available libraries. Each acquired Raman spectrum was preprocessed in the following order: baseline subtraction, smoothing and rescaling. First, the improved asymmetric least-squares (IAsLS) method from pybaselines library was used to subtract the baseline. Second, data was smoothed by Savitzky–Golay filter from scipy. Lastly, to prepare data for statistical analysis, rescaling was performed with the standard scaler from sklearn. After preprocessing the data was dimensionally reduced with principal component analysis (PCA) and a model was trained with a K-Neighbors Classifier, both from sklearn library.

## Results and Discussion

### Synthesis, Optical, Physical, and Chemical Characterization

For the Au^3+^ to Au^0^ reduction, all samples were exposed to CAP ignited between the solution surface and the gold wire electrode (Figure 1a). The discharge was maintained with a sinusoidal voltage of 12.5 kV peak-to-peak at 20 kHz (Figure 1b), with a nominal plasma power of 5.4 ± 0.2 W calculated by averaging the product of the current and voltage waveforms over multiple cycles.^34^ OES revealed the plasma’s characteristics during AuNP formation, showing emission lines of argon, hydroxyl, atomic hydrogen, and atomic oxygen (Figure 1c). Notably, nitrogen emissions (300–450 nm) were absent, indicating complete air displacement from the vial due to a continuous 1500 sccm argon flow. The presence of hydroxyl radicals (309 nm), hydrogen Balmer lines (486.1 and 656.3 nm), and atomic oxygen (777 nm) was attributed to water in the reactor.

The gas temperature, a critical parameter in plasma-assisted processes, was estimated at *T*_rot_ ≈ 590 K using rotational temperature simulations of OH emission profiles (306–312 nm) with LIFEBASE software (Figure 1d).^35,36^ Despite minor spectral discrepancies, the derived temperature correlated well with the experimental data. Free electrons in the plasma are assumed to play a pivotal role in reducing Au precursors into nanoparticles, with their density estimated at *n*_e_ ≈ 4.38 × 10^16^ cm^–1^ via Stark broadening of the H_β_ line at 486.1 nm. The Stark broadening was determined using a Voigt profile to separate the Lorentzian (Stark, van der Waals) and Gaussian (instrumental) components, excluding resonance broadening due to its negligible effect.^37−39^

Convection and mixing by the argon flow moderated thermal effects, with only a slight solution temperature rise from 22 to ∼36 °C after treatment. This suggests that the solution temperature had minimal influence on the reduction process.

**Figure 2:**
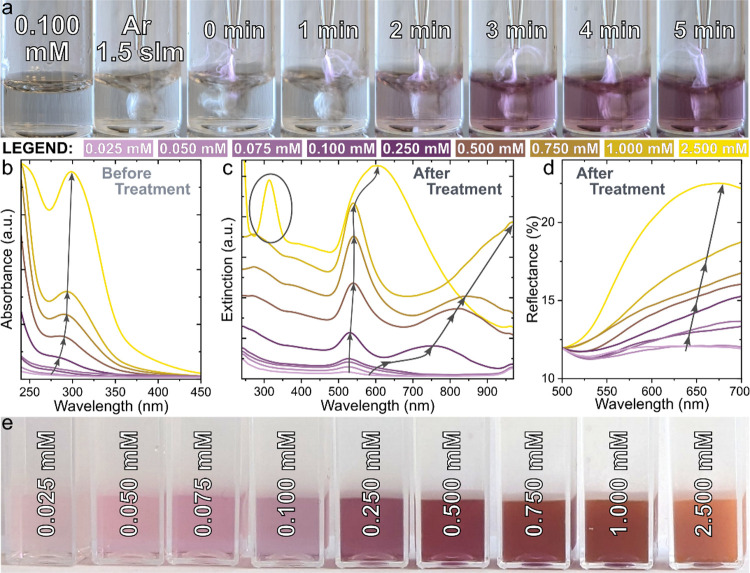
(a) Photographs showing the progression of nanoparticle synthesis from aqueous precursor solution to colloidal nanogold over 5 min of treatment time for the 0.1 mM concentration case; (b) UV–vis absorbance spectra of the stock solution indicating Au^3+^ peak intensity with concentration; (c) extinction spectra of colloidal nanogold showing an increase in the surface plasmon resonance peak intensity with concentration; (d) total diffuse reflectance of the colloidal nanogold; (e) colloidal nanogold after plasma treatment with increasing precursor concentration from left (0.025 mM) to right (2.5 mM).

Figure 2a highlights the progression of nanoparticle formation from the gold precursor (0.1 mM) at different treatment times, where a characteristic color is first observed after 2 min, indicating AuNP formation. Other concentrations are highlighted in the Supporting Information (Figure S1). After 5 min of treatment, the solution color had intensified to violet for the 0.1 mM sample. UV–vis absorption measurements were performed before and after treatment. Absorption measurements of the untreated precursor show a clear peak from 270–300 nm, which typically represents nonreduced gold ions, and grows with increased precursor concentration (Figure 2b). Following plasma exposure, the peak associated with gold ions was observed to disappear, except for the 2.5 mM solution, indicating only partial reduction of the precursor material (Figure 2c). In contrast, a new characteristic plasmon peak for Au nanoparticles appeared at 520 nm, which gradually shifted to higher wavelengths and became broader as the concentration increased. The red shift can be attributed to an increase in the size of spherical nanoparticles, confirmed using SEM and TEM. Further, a wide hump was observed for the 0.25–1.0 mM samples, which is indicative of the presence of planar nanogold shapes. The diffuse total reflectance spectra (Figure 2d) indicate an increased contribution of the scattering component, which is typically observed from larger nanoparticles and plate-like geometries. Following Ar plasma treatment, all stock solutions (0.025, 0.05, 0.075, 0.1, 0.25, 0.5, 0.75, 1.0, and 2.5 mM) changed color from clear/pale-yellow to pink/reddish and acquired specific transparency/scattering features (Figure 2e). The lowest concentration sample was pale pink and optically transparent (negligible reflectance). The transparency of the colloidal solution reduced at higher concentrations, which can be clearly seen in the 1.0 mM sample. After 5 min of treatment, the 2.5 mM sample featured thin gold flakes floating on the surface with some sedimented at the bottom (Figure S2), and under such conditions, particles appeared as fused irregular agglomerates, suggesting the limit of synthesis control.

**Figure 3:**
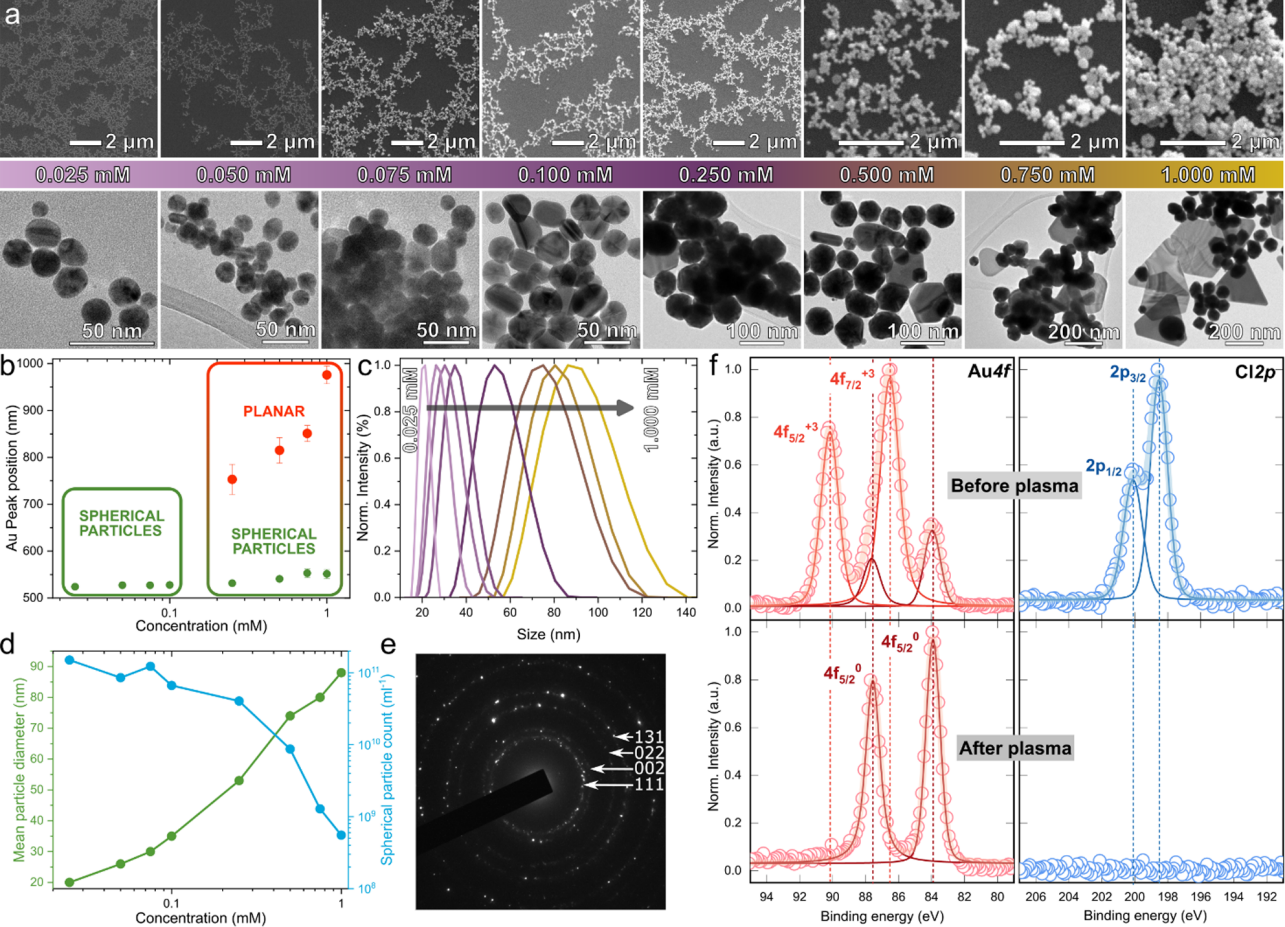
(a) Series of SEM and TEM images showing the size and shape of Au nanoparticles with increasing concentration of starting material; (b) Au^0^ peak positions in UV–vis extinction spectra. (c) DLS/MALDS measurements of 0.025 to 1.0 mM nanocolloids and their (d) spherical size and count distribution as a function of precursor concentration; (e) selected area electron diffraction (SAED) ring patterns for nanocolloids created using a 1.0 mM precursor concentration; and (f) deconvoluted XPS spectra showing the Au 4f and Cl 2p core levels for the 1.0 mM sample before and after CAP treatment.

As shown in Figure 3a, the size of the spherical AuNPs increased from approximately 20 nm at 0.025 mM to 90 nm at 1.0 mM. At lower concentrations, especially between 0.1 and 0.25 mM, the AuNP shapes were mostly of the multiply twinned geometry (5-fold rotational symmetry, regular and irregular decahedron, icosahedron) with little or no inclusion of plate-shaped nanogold (2-fold and 3-fold twin crystal).^40^ From 0.25 mM and above, especially in the case of 1.0 mM, plate geometries (trigonal and hexagonal plates) were pronounced. Interestingly, on closer inspection of the UV–vis absorbance data (Figure 2c), two peaks are observed in the same samples, indicating a mixture of plates and spherical shapes (Figure 3b). From DLS, apart from a spherical particle size increase, it was observed that the size distribution increased with increasing precursor concentration. The associated size of spherical AuNPs increases monotonically (Figure 3c), while the opposite occurs for their concentration (here spherical shapes are considered only), which drops from 10^11^ particles/ml at 0.025 mM down to 5 × 10^8^ particles/mL at 1.0 mM, as shown in Figure 3d. There is a clear window for the production control of spherical particles solely, which is highlighted in Figure 3b. For particles created using 1.0 mM precursor concentration, it was possible to estimate the size (∼300 nm) and also the concentration (2 × 10^7^ particles/mL) of plate-like geometries.

Typically, the formation of thin prisms is associated with a decrease in pH and/or an increased concentration of noble metal cations.^41^ In analogy to the thin Ag nanoplatelets of Zhang et al., selective formation of nanoparticles with a higher anisotropy defined by (111) facets can be achieved at higher Ag^+^ content and low pH by preferential deposition on (100) facets of planar twinned seeds.^42^ Critically, under the conditions investigated, the Au^3+^ concentration was increased, and the pH was reduced as the plasma treatment progressed. Also, pH was lower for higher chloroauric acid content in water, as confirmed by measurements in Table S1. At low Au^3+^ concentrations (0.025–0.1 mM), the entire precursor was rapidly consumed (i.e., the color changed after 2 min of exposure with no further change), whereas with the 0.25–1.0 mM samples, a transparent pink color was initially observed which evolved throughout the treatment, indicating the emergence of distinct scattering features. The formation of high aspect ratio shapes and plate-like shapes is consistent with an increased scattering profile in the UV–vis region >600 nm and agrees with microscopy data. Focusing on the 1.0 mM sample, the complex SAED ring pattern suggests the presence of polycrystalline nanogold (Figure 3e).

The reduction efficiency was tested via XPS (Figure 3f) by tracing the Au^3+^ surface component contribution and the chlorine content. Prior to treatment (Figure 3f, red curves, top), the spectra predominantly featured Au^3+^ peaks, accompanied by less pronounced Au^0^ components. The two peaks at binding energies of 90.1 and 86.4 eV are consistent with Au^3+^ (from the [AuCl_4_]^−^ complex) as well as two at 87.7 (4*f*_5/2_) and 84.0 eV (4*f*_7/2_) assigned to Au^0^.^43^ Following plasma exposure, the spectrum exhibited only Au^0^ peaks, suggesting a complete reduction of the ionic part of the gold precursor. To further verify this, the Cl 2*p* region was examined (Figure 3f, blue curves). Initially, a distinct doublet was observed with binding energies of 200.1 eV (2*p*_1/2_) and 198.6 eV (2*p*_3/2_), which was clearly absent in the treated sample. Similarly, no evidence of the chlorine Kα line at 2.62 keV was detected from EDS for the same 1.0 mM concentrated sample (Figure S3).

### Reproducibility, SERS Enhancement, Detection Limit

Due to poor reduction control, the 2.5 mM sample was ruled out for SERS testing. |With the 1.0 mM sample being chosen for further experimentation owing to its pronounced extinction profile at 633 nm. First, 3 consecutive butches of 1.0 mM were prepared (Figure 3a,) and concentrated via centrifuging. the resulting solution was mixed with crystal violet CV (10^–6^ M), followed by drop-deposition and drying on Si for the analytical enhancement factor (aEF) study.

**Figure 4:**
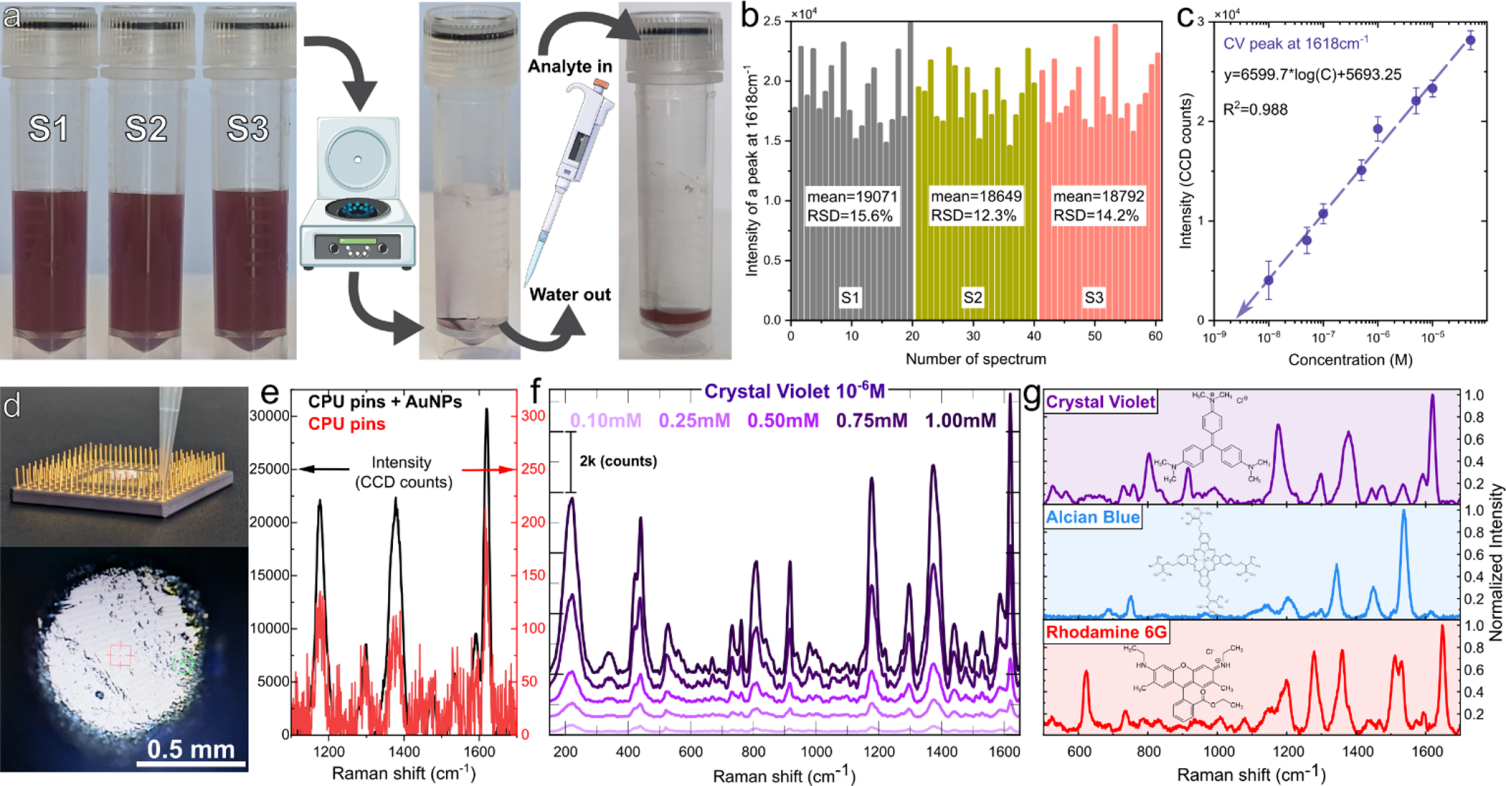
(a) Process diagram showing three independent samples of nanogold created using 1.0 mM precursor, centrifuged and the analyte added for SERS measurements; (b) corresponding spectra of CV (10^–6^ M) recorded from dried spots on Si wafer each accompanied by relative standard deviation measurements; (c) detection limit estimation taking intensity profile of a mode located at 1618 cm^–1^ from the SERS concentration study of crystal violet Figure S5; (d) deposition of a mixture of water diluted Raman molecule (CV:10^–6^ M) with nanoparticles on CPU pins; (e) SERS comparison of CV (10^–6^ M) for CPU pins with Au nanoparticles “black” and CV (10^–6^ M) without Au nanoparticles “red” curve; (f) SERS test of nanocolloids obtained from different Au^3+^ precursor concentrations; (g) SERS fingerprints (10^–6^ M) of crystal violet, alcian blue, and rhodamine using 1.0 mM AuNPs.

Compared to bare silicon (Figure S4, CV concentration was 10^–1^ M, peak at 1618 cm^–1^ as reference), the average analytical enhancement factor (aEF) at coffee ring hotspots, where the performance is known to be better,^44,45^ was estimated to be aEF ≈ 2.5 × 10^7^. For the aEF estimation, a modified formula was used taking into consideration a different acquisition time and analyte concentration , where *I*_SERS_ and *I*_RS_ are the experimental Raman intensities from nanogold on Si and from bare Si; *P*_SERS_ and *P*_RS_ are the laser power, which in our case was held constant (1.5 mW); *T*_SERS_ and *T*_RS_ are the acquisition times (2 s for SERS and 10 s for Raman); and *N*_SERS_ and *N*_RS_ are the CV concentrations.^46^ The relative standard deviation of the corresponding nanogold samples S1–S3 was within a range of 12–16%, demonstrating performance stability (Figure 4b), which correlates with the repeatability of the UV–vis spectra (Figure S5), where minor deviations at >750 nm may be related to slight deviations in plate-like NP concentrations. The detection limit (AuNPs on Si) was estimated by extrapolating a linear fit of the ν(C=C) intensities in the 1618 cm^–1^ mode and is estimated to be in the range of 1 × 10^–9^ M (Figures 4c, and S6).

Also, it is known that SERS from NPs can be greater if, instead of using a Si support, a metal surface is used, due to so-called “third-generation hot spots” originated as a result of the hybridization of the scattered (from NPs) and reflected (from substrate) EM fields.^47^ As described in Ding et al., an identical plasmonic NP placed on Pt can generate up to 1 order of magnitude higher signal compared to a Si substrate.^48^ To test this scenario, a droplet containing concentrated AuNPs and CV was deposited on top of a gold-plated CPU pin-grid array. Comparing the intensities from AuNPs on Si and AuNPs on Au-coated pins (Figures 4d,e, S7), an additional 60–70% SERS gain is obtained due to plasmonic coupling between the nanogold and the Au coating of the pin, while bare pins (microstructured surface) provide an aEF of about 5.6 × 10^4^, which is typical for flat Au films.^49^ The performance of other AuNP samples was also compared, with SERS intensity ratios as follows: *I*(_1.0 mM/0.1 mM_) = 43 ± 2; *I*(_1.0 mM/0.25 mM_) = 9.9 ± 0.6; *I*(_1.0 mM/0.5 mM_) = 4.5 ± 0.5; and *I*(_1.0 mM/0.75 mM_) = 1.8 ± 0.3, indicating the greater performance of the sample containing a combination of both: large size 5-fold truncated particles and planar shapes (Figure 4f) resulting in additional plasmonic coupling conformations, including plate–plate, sphere–sphere, and plate-sphere forming hot-spots and especially hot-lines as demonstrated by EM distribution modeling via COMSOL software (Figure S8). The 1.0 mM sample was used to record other fingerprints from Raman markers alcian blue and rhodamine at 10^–6^ M (Figure 4g), demonstrating versatility for other analytes that differ in molecular structure, charge-transfer mechanism, and adsorption efficiency to metal nanostructures.^50^

### SERS of Explosives

**Figure 5:**
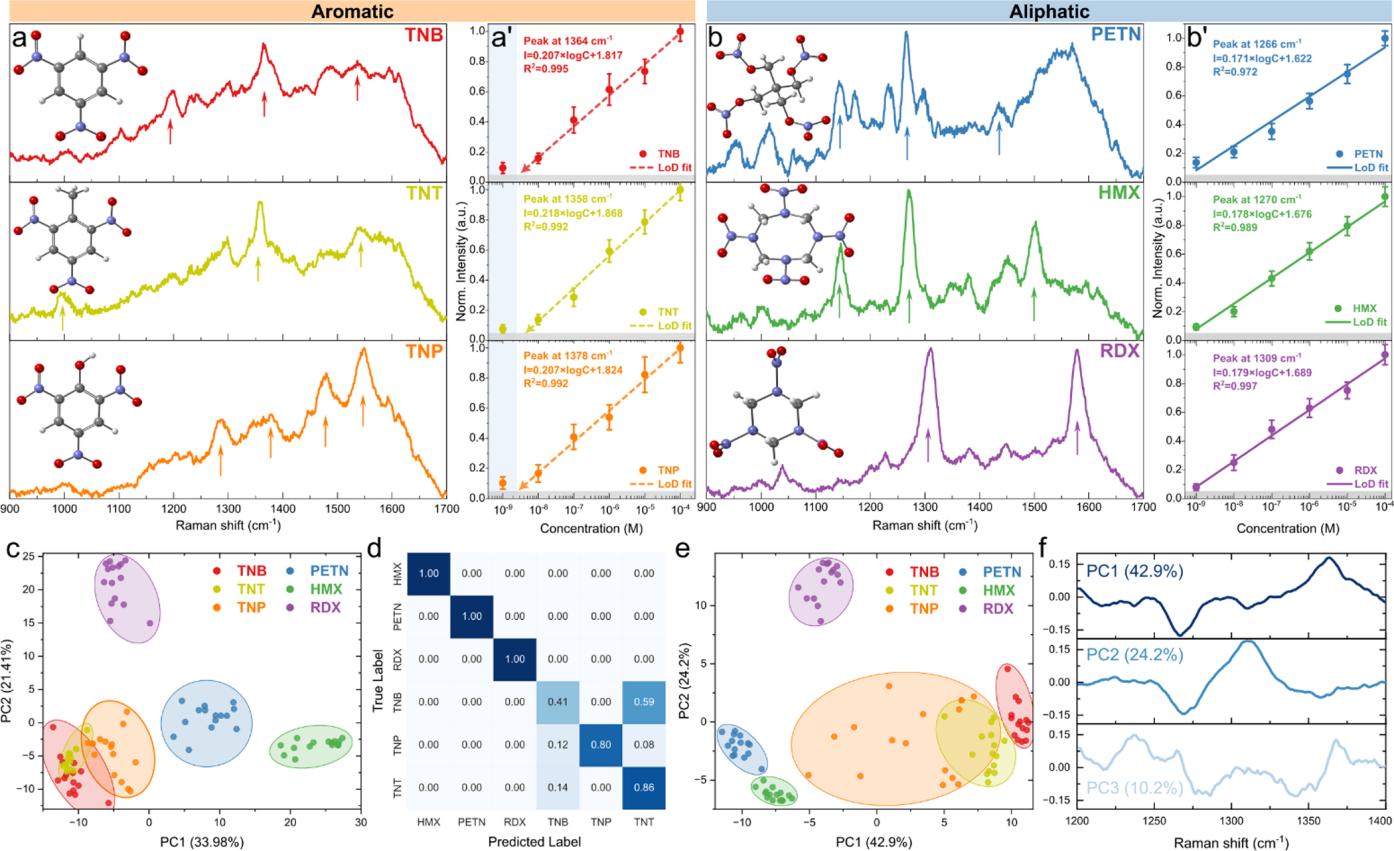
(a) SERS of the aromatic TNT, TNP, TNB and (b) aliphatic RDX, HMX, PETN explosives after baseline correction with corresponding (a’, b’) LoD fitting; (c) PC1 vs PC2 and its evaluation with (d) k-NN confusion matrix (up to PC3) within a wide-range Raman spectrum (750–1700 cm^–1^) of aromatic and aliphatic explosives; (e) PC1 vs PC2 and (f) corresponding loading plots of ν_s_(NO_2_) band (1200–1400 cm^–1^).

To demonstrate the utility of the approach for sensing trace compounds, six high-energy molecules, including three aromatic molecules (TNT, TNP, and TNB) and three aliphatic (RDX, HMX, and PETN), were SERS tested, focusing on RSD behavior and detection limit. The typical fingerprints are shown in Figure 5a,b. For clarity, the presented spectra are averaged and normalized, while raw SERS data are presented in Figure S9.

The profiles of the aromatic molecules (TNT and TNB) are quite similar due to the features of the ring structure and nitro group configurations, with minor differences caused by the methyl group (TNT). The third representative TNP, also known as picric acid, contains an additional OH and C–O bond that makes the spectra more complex. Regardless, all three compounds feature a distinct peak between 1320 and 1370 cm^–1^, characteristic of ν_s_(NO_2_) in nitro-aromatic compounds. Also, a contribution from C=C and C–H vibrations are observed (1400–1700 cm^–1^). The aliphatic molecules RDX and HMX reveal sharper modes in the fingerprint range; these two compounds consist of six- and eight-membered rings, respectively, with alternating carbon and nitrogen atoms. Their ν_s_(NO_2_) signal is somewhat lower compared to nitro-aromatic molecules, a finding that agrees with data from the literature in the 1280–1340 cm^–1^ range.^51^ The PETN aliphatic molecule has no ring, but the Raman profile is richer due to the contribution and possible overlap of C–O and C–C and C–H_2_ stretching and bending vibrational components. This molecule belongs to a class of nitrate esters and is characterized by ν_s_(NO_2_) in the range 1250–1300 cm^–1^. All of the main vibration characteristics are consistent with or very similar to those reported in the literature, taking into account the laser wavelength of the SERS probing. For instance, the ν_s_(NO_2_) mode of the tetryl molecule appears at approximately 1329 and 1357 cm^–1^ with 532 and 785 nm laser excitation, respectively.^52,53^ Further, the sensitivity performance and signal stability were tested by means of a limit of detection (LoD) study (Figures 5a’b’, S10, intensities vs concentrations) and relative standard deviation behavior. Inspecting both the aromatic and aliphatic compounds, it can be suggested that the limit of detection for TNB, TNT, and TNP (picric acid) is in a range of ∼2–6 nM, while for aliphatic compounds HMX, RDX, and PETN, the LoD enters to the subnanomolar (∼600–900 pM) region, aligning well with the best-performing substrates reported for nanoparticles-based substrates.^54−59^ Note, the point corresponding to the lowest concentration for aromatic compounds was omitted from the fit as it significantly reduced *R*^2^ below 0.970 while overestimating the LoD, creating fantom signals in the ultralow concentration regime.^60^ Contrary, for aliphatic compounds, a linear trend was observed with *R*^2^ > 0.970 down to the lowest concentration measured. As a result, the LoD slopes for HMX, RDX, and PETN (0.17–0.18) are smaller than those for TNT, TNB, and TNP (0.20–0.21). During testing, TNP was found to be highly unstable, making it barely usable for detection under the same conditions as the other molecules (Figure S11). However, the ν_s_(NO_2_) range (around 1370 cm^–1^) showed the most consistent performance in terms of RSD behavior compared to other modes (Figure S12). Notably, a literature survey reveals a strong discrepancy of TNP fingerprints among studies, where in some cases, the typical NO_2_ symmetric stretching range is more silent than secondary peaks, which may be a sign of molecule decomposition during analysis.^61−64^ Comparing SERS to an alternative optical method, namely, UV–vis absorbance, commonly employed in forensic practice, the latter exhibits significantly poorer performance, barely reaching a 10^–4^–10^–5^ detection limit as demonstrated for TNB molecules by monitoring their characteristic UV absorption peak at 230 nm.^65−67^ (Figure S13).

Moving deeper into a data mine, the RSD in general is found to be smaller (Figure S11, 10^–6^ M case) for aliphatic compounds (RSD ∼ 6–10%) than for aromatic molecules (RSD ∼ 12–17%). Naturally, the RSD becomes higher at lower concentrations, and its progression is much faster for aromatic molecules TNT, TNB, and TNP (Figure 5a’,b’). Such a situation could be related to the stability of the compounds under the given excitation conditions (photostability and thermal stability), linking also to their vapor pressure properties. In general, the lower the pressure, the more stable the molecule (for HMX, RDX, and PETN ∼10^–8^–10^–11^ atm, while for TNT, TNB, and TNP, this number is about 10^–5^ atm at ambient temperature).^66,68,69^ From the literature, it is known that the aromatic compounds are less stable than aliphatic molecules, where the tested analytes can be arranged in an approximate order from most to least stable explosive molecules HMX → RDX → PENT → TNT → TNB → TNP, corresponding to our RSD observations.^70−72^

The poorer LoD and RSD values of the aromatic TNB, TNT, and TNP molecules can affect their classification using common statistical and dimensionality reduction methods such as PCA. To examine this, two simple cases of PCA were examined where the selection of a “region of interest” can facilitate recognition of structurally similar explosives such as TNT, TNB, and TNP. In the first scenario, a wide range of 750–1700 cm^–1^ was used. In Figure 5c, three well-separated clusters are observed at 10^–6^ M representing the aliphatic HMX, RDX, and PETN explosives. However, no conclusion regarding the specific analyte type can be drawn based on the first two PCs, due to higher RSD fluctuation and similarity of vibrational spectra, especially for TNT and TNB. Training the k-nearest neighbors (k-NN) algorithm (process depicted in Figure S14) on PCA dimensionally reduced wide-range Raman spectra quantifies the lack of separation of the aromatic explosive molecules. The inclusion of a higher-order PC (between 80 and 90% of variance coverage) in the training data set improves the separation above 95%. However, a similar outcome can be achieved with a refined focus on the characteristic ν_s_(NO_2_) band considering mode positions for nitro-aromatic, nitro-amines, and nitro-esters (1200–1400 cm^–1^). Similar to the wide-range case, the separation of aliphatic explosives into the respective clusters can be seen; moreover, decoupled clusters of TNT and TNB appear alongside a larger smear of TNP points caused by the high RDS in SERS data (Figure 5e). Loading plots, for the narrow band, in Figure 5f indicate that the nitro-aromatic range (1350–1375 cm^–1^) dominates the PC1 contribution, while PC2 further helps distinguish between aliphatic explosives. This method proves to be effective when only these six explosives are compared, which exhibit distinct and well-defined features in their Raman spectra. The PCA approach in combination with the k-NN algorithm allows a clear distinction based on the characteristic spectral patterns of each analyte. However, if the analysis is to be extended to a wider range of analytes or if the aim is to discriminate compounds without prior knowledge of their Raman profiles, a PCA-kNN approach with a wider spectral range would be more advantageous. By including a wider spectral range, the model can capture more subtle differences between unknown analytes, improving its robustness and versatility in real-world applications.

## Conclusions

In summary, we demonstrate that Raman scattering can be significantly enhanced by the inclusion of planar nanogold synthesized using an efficient and single-step plasma-liquid redox approach which offers a clean, fast, and green process. In liquid, rapid reduction involving no external chemical reagents offers a control in the size of spherical AuNPs as well as the increased outcome of planar shapes by simply increasing the concentration of the Au^3+^ precursor. The plasmonic optical response tested by modeling and Raman marker experiments of the sphere–sphere, sphere-plate, and plate–plate field confinement conformations in the designed Au nanoparticle mix was used to detect multiple trace explosives down to 5 × 10^–9^–6 × 10^–10^ M concentrations, with nearly 4 orders of magnitude better sensitivity observed over the widely used UV absorption spectroscopy approach. Through the simple and efficient combination of microplasma synthesis and reuse of a Au-coated pin grid array of a CPU, we developed a novel plasmonic-based sensor for sensitive SERS measurements that is highly effective for probing low concentrations of hazard molecules. Additionally, the discrimination of explosives can be improved either with analysis augmented by the predictive machine learning PCA-k-NN method or by focusing on the specific NO_2_ fingerprint range, addressing the signal fluctuation differences between aliphatic compounds (RSD ∼ 6–10%) and their volatile aromatic counterparts (RSD ∼ 12–17%). With a comprehensive Raman database for explosives, the SERS-PCA-kNN combined with nanogold plasmonic sensors can be a powerful analytical tool for detecting explosives in aqueous solutions.

## Supporting Information Available

Additional experimental details for plasma characterization with OES measurements of a discharge, gas temperature modeling using Balmer lines, nanoparticles characterization including SEM, UV–vis, digital photos for in situ visual inspection, and EDS data; modeling supporting electric field confinements in different conformations; and tables containing featuring Raman modes of the explosives tested, table for PCA + K-NN optimization and performance metrics, and UV–vis data for TNB molecule (PDF)

## Abbreviations

SEF: surface enhanced fluorescence
SEIRA: surface enhanced infrared absorption
SERS: surface enhanced Raman scattering
DBD: dielectric barrier discharge
CPU: central processing unit
SEM: scanning electron microscope
TEM: transmission electron microscope
TNT: trinitrotoluene
TNP: trinitrophenol
TNB: trinitrobenzene
HMX: high melting explosive
RDX: research department explosive
PETN: pentaerythritol tetranitrate
PCA: principal component analysis
k-NN: k-nearest neighbors
