# Supplementary material for: Microplasma Controlled Nanogold Sensor for SERS of Aliphatic and Aromatic Explosives with PCA-KNN Recognition
Source: ACS Sens. 2024 Dec 24;10(1):387–97. doi: 10.1021/acssensors.4c02651 (PMC11773561; doi:10.1021/acssensors.4c02651)
Supplement: Supplementary file 1 — se4c02651_si_001.pdf [file se4c02651_si_001.pdf]

Microplasma Controlled Nanogold Sensor for SERS of Aliphatic and Aromatic Explosives with PCA-KNN Recognition

Jaka Olenik[1,2] ‡, Vasyl Shvalya[2,] ‡, Martina Modic[2], Damjan Vengust[2], Uroš Cvelbar[2], James L. Walsh[1,2]*.
Corresponding author: James L. Walsh (james.l.walsh@york.ac.uk)

[1] York Plasma Institute, School of Physics, Engineering and Technology, University of York, York YO10 5DD, UK
[2] Department for Gaseous Electronics F6, Jozef Stefan Institute, 1000 Ljubljana, Slovenia 1000 Ljubljana, Slovenia
KEYWORDS: Nanogold Scatterers, Plasma Synthesis, SERS, Explosives, Machine Learning.

Supporting information

1. PLASMA CHARACTERISATION
2. FORMATION OF AU NANOPARTICLES VIA COLOUR CHANGE
3. PHOTO AND SEM OF 2.5 MM SAMPLE AFTER TREATMENT
4. PH MEASUREMENTS OF A AU PRECURSOR DISSOLVED IN WATER
5. EDS MEASUREMENTS
6. RAMAN OF CRYSTAL VIOLET DROPLET ON SI WAFER
7. OPTICAL PROPERTIES OF BATCH-TO-BATCH NANOGOLD FOR A SELECTED CONCENTRATION
8. SERS OF CRYSTAL VIOLET
9. SEM OF AUNPS ON CPU PINS
10. COMPUTATIONAL ELECTROMAGNETIC MODELING
11. RAW SERS DATA FOR EXPLOSIVES
12. LITERATURE SURVEY OF RAMAN ASSIGNMENTS FOR EXPLOSIVES
13. SERS OF EXPLOSIVES: DATA FOR LOD STUDY
14. RELATIVE STANDARD DEVIATION
15. SERS VS UV-VIS LIMIT OF DETECTION
16. PCA+K-NN OPTIMIZATION
17. REFERENCES

1. PLASMA CHARACTERISATION

Using a Voigt profile, as shown in Figure 1e, the Lorentzian and Gaussian broadening contributions were obtained. The main contributor to Gaussian full width at half maximum (FWHM) is the instrumental function of the spectrograph. While the Lorentzian component consists of Stark, Van der Waals and Resonance broadening, the latter is insignificant and can be excluded from calculation.[1] The Van der Waals broadening was calculated using the following formula (1).[2]

$$\Delta\lambda_{vdW} = \frac{4.1}{T_g^{0.7}} \quad (1)$$

Where T_g is the gas temperature that was previously estimated to be 590 K.

The remainder of the broadening can therefore be attributed to Stark broadening. The electron density n_e was estimated to be 4.38 x 10^{16} cm^{-1} from the Stark broadening using the following formula (2).[3]

$$n_e = 10^{17}\left(\frac{\Delta\lambda_S}{0.48}\right)^{1.46808} \quad (2)$$

During interaction with the water surface, energetic electrons in the plasma initiate a cascade of chemical processes that generate reactive species at the liquid interface, yielding solvated electrons, O_3, O_2, O, OH, HO_2, O_2^- and H_2O_2, which act as reducing agents for Au^{3+}.[4]

2. Formation of Au nanoparticles via colour change

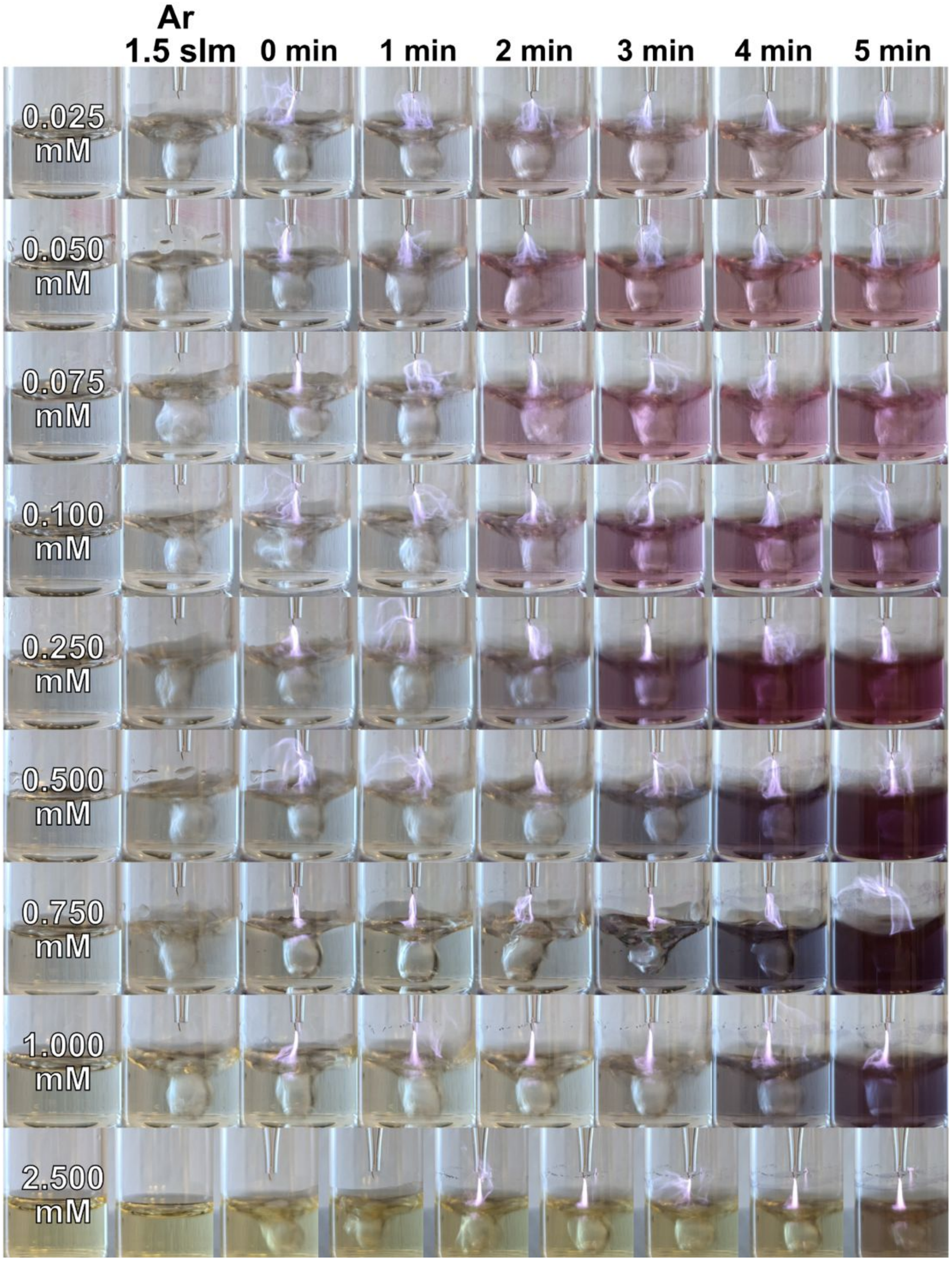

Figure S1: Process of the nanoparticle synthesis from aqueous precursor solution to colloidal nanogold over 5 min of treatment time

3. Photo and SEM of 2.5 mM sample after treatment

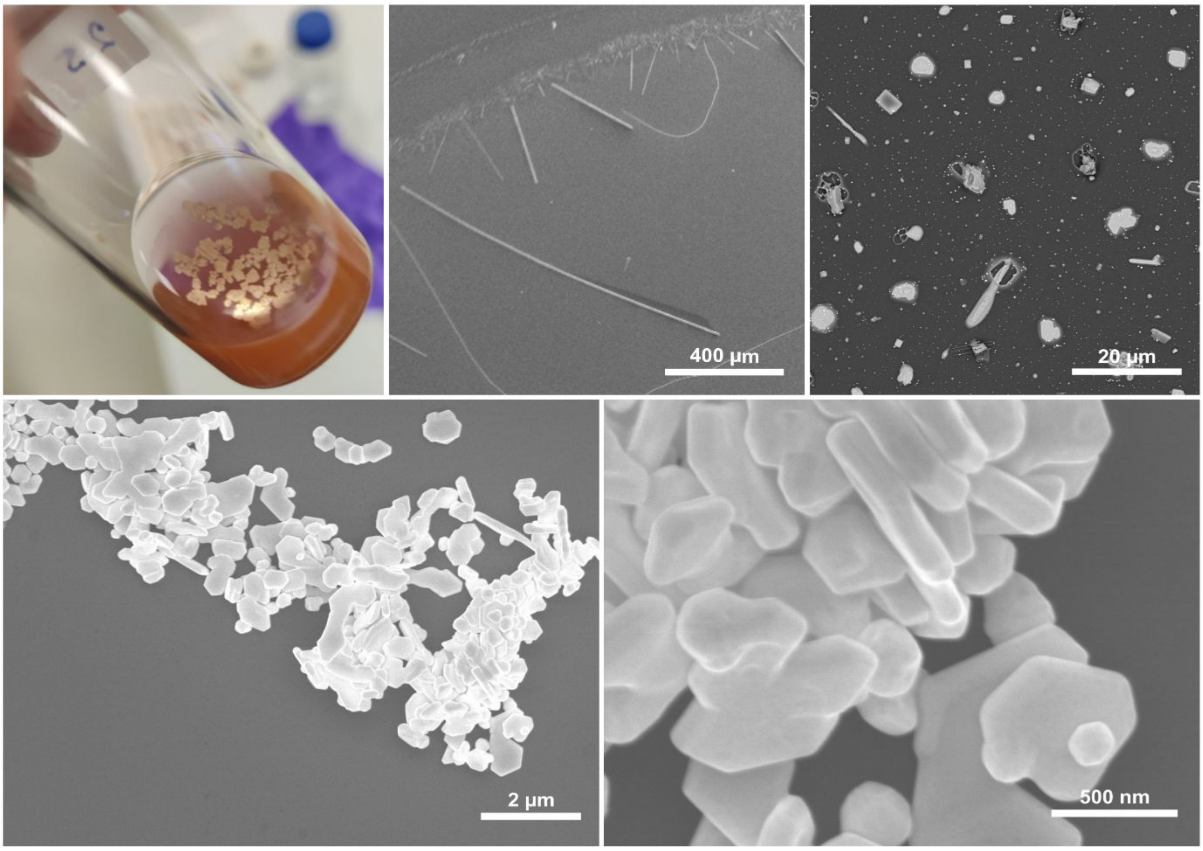

Figure S2: Digital photo of a 2.5 mM sample immediately after the treatment revealing gold flakes, accompanied with SEM micrographs showing AuNPs and unreduced precursor salts forming lines of solid $AuCl_3$ at coffee-ring placed and in centre of a dried droplet.

4. pH measurements of a Au precursor dissolved in water

After centrifuging aqueous $HAuCl_4 \cdot 3H_2O$ solutions the measurements of pH were taken with a handheld pH meter (HI-98103, HANNA INSTRUMENTS).

Table S1. pH values of precursor solution

Precursor solution concentration [mM]	pH value
0.025	4.8
0.050	4.1
0.075	4
0.100	3.9
0.250	3.5
0.500	3.2
0.750	3.1
1.000	3
2.500	2.8

5. EDS MEASUREMENTS

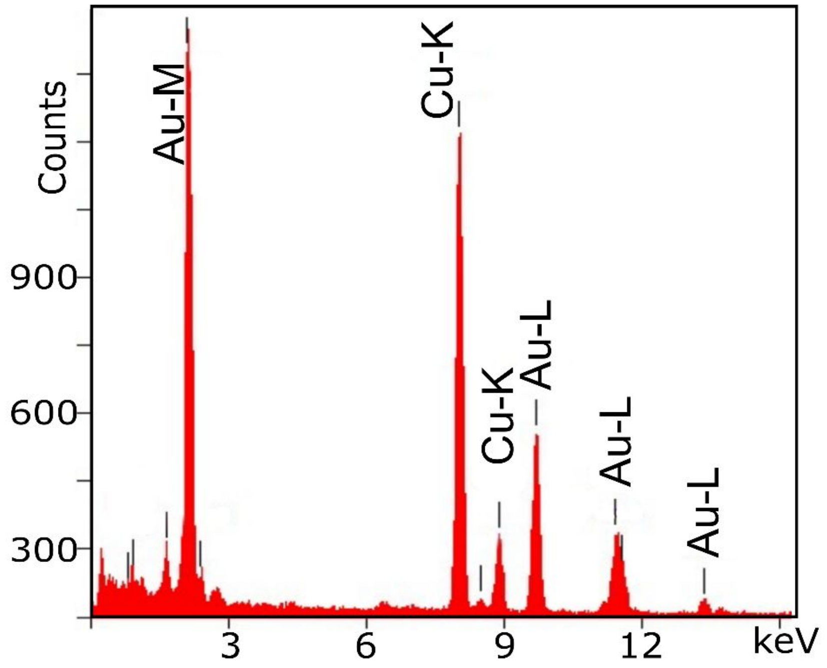

Figure S3: Energy dispersive X-ray spectroscopy (EDS) for 1.0mM nanocolloids

6. RAMAN OF CRYSTAL VIOLET DROPLET ON SI WAFER

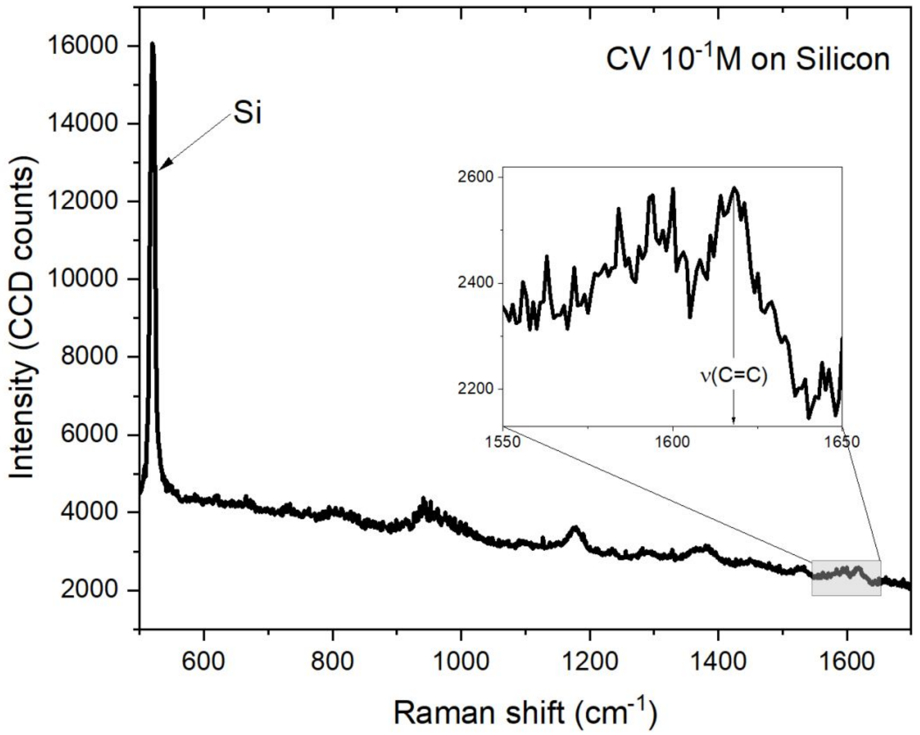

Figure S4: Raman of crystal violet droplet dried on Si wafer. An RMS20X - 20× Olympus Plan Achromat objective, 0.4 NA, 1.2 mm WD was used for the investigation, 633nm He-Ne laser, exposure time 10s.

7. Optical properties of batch-to-batch nanogold for a selected concentration

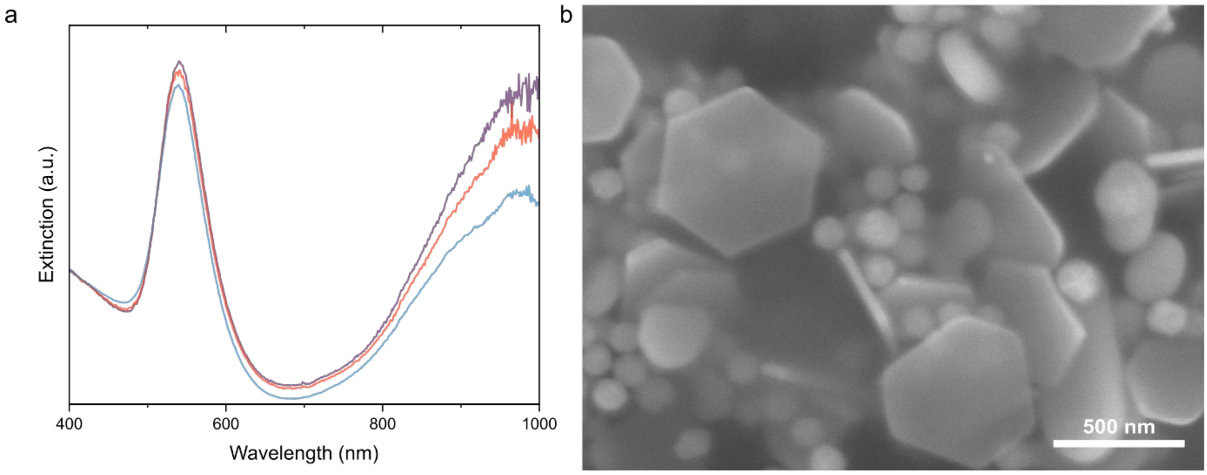

Figure S5: (a) Extinction spectra of 1.0 mM solution from different treatment batches and (b) their SEM image of the AuNP

8. SERS of crystal violet

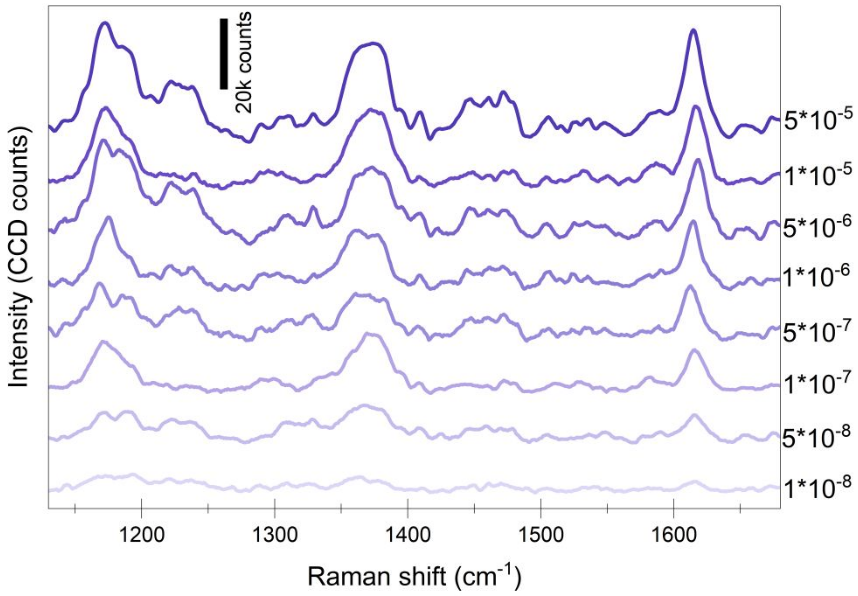

Figure S6: SERS concentration study of crystal violet on Si with nanoparticles. An RMS20X - 20× Olympus Plan Achromat objective, 0.4 NA, 1.2 mm WD was used for the investigation, 633 nm He-Ne laser, exposure time 2s.

9. SEM OF AUNPS ON CPU PINS

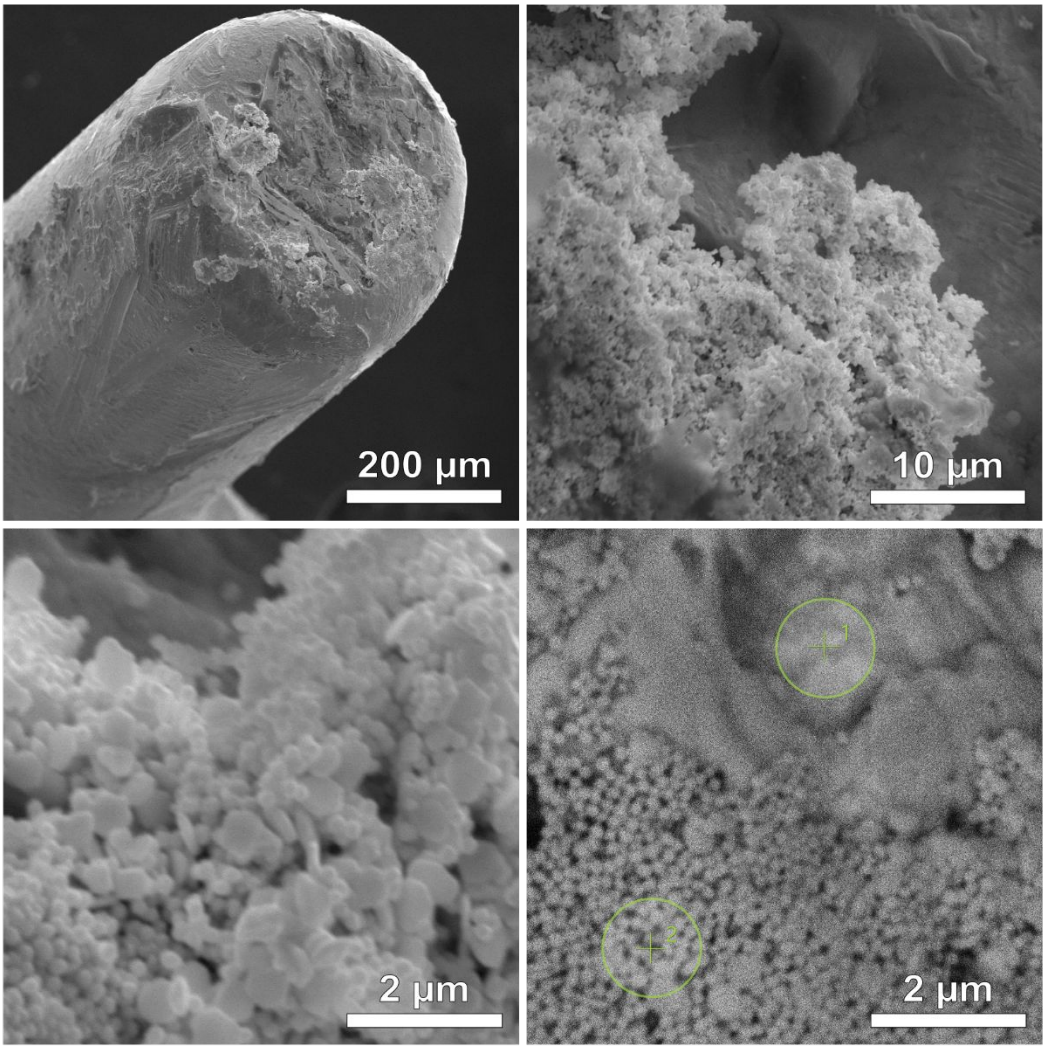

Figure S7: SEM of the CPU pins covered with AuNPs with EDS of CPU pin at point 1 and EDS of AuNP on the CPU pin at point 2

Table S2: EDS of CPU pin at point 1

Element	Atomic %	Atomic % Error	Weight %	Weight % Error
C	32.3	2.1	3.7	0.2
O	18.1	2.3	2.8	0.3
Au	49.7	0.6	93.5	1.1

Table S3: EDS of AuNP on the CPU pin at point 2

Element	Atomic %	Atomic % Error	Weight %	Weight % Error
C	28.8	1.8	2.4	0.1
Au	71.2	0.9	97.6	1.2

10. COMPUTATIONAL ELECTROMAGNETIC MODELING

Computational simulations by COMSOL emphasize the relevance of side-by-side coupling of thin trigonal or/and hexagonal prisms for optimal analytical performance. From the results it is clear that the line coupling (2D-2D) outperforms the point coupling (3D-3D) offering substantial field enhanced over a large area for analyte excitation.

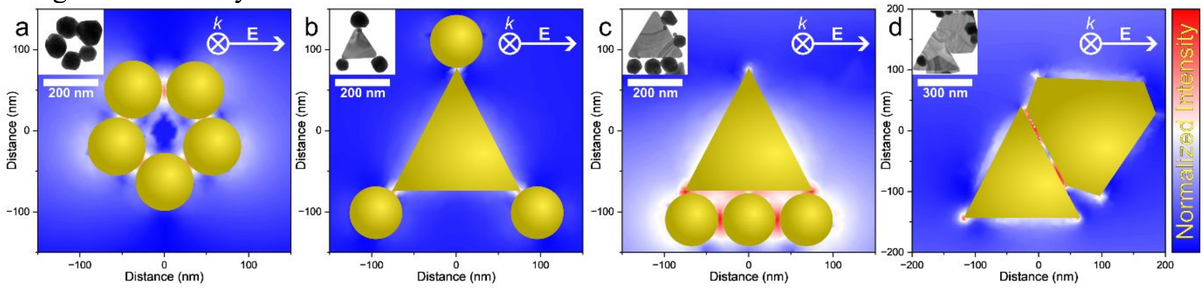

Figure S8: Illustration of the most common electric field confinement regimes (using a normalized scale) for (a) 3D-3D, (b,c) 3D-2D and (d) 2D-2D gold nanoparticles conformation that can be observed for a sample prepared from 1.0 mM Au^{3+} aquatic solution.

11. Raw SERS data for explosives

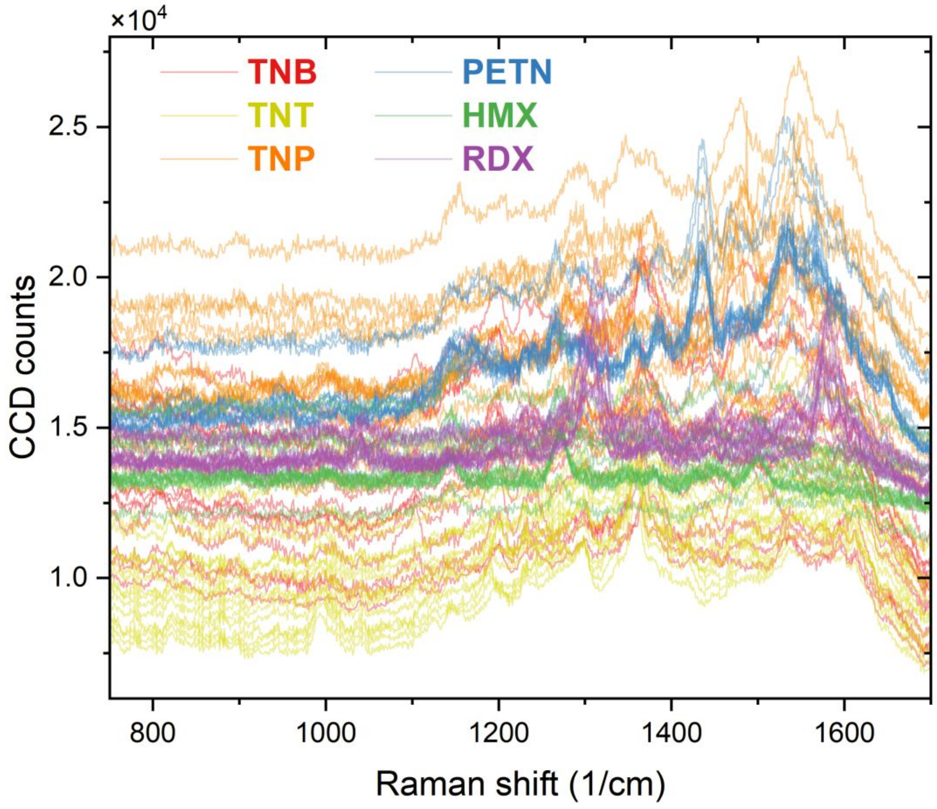

Figure S9: Raw data for explosives with 15 raw spectra for each explosive

12. Literature survey of Raman assignments for explosives

Table S4: Published Raman band assignments for explosives.

Raman Shift [cm^{-1}]	Assignment	Ref
793	C–H out-of-plane bend	[5,6]
826	Aromatic ring deformation	[6]
878	symmetric ring-breathing mode	[7,8]
932 - 996	ring stretching	[7,8]
940	C−N stretching	[5]
1026	CH_3 deformation	[5]
1074	C–C stretching	[8,9]
1142	C–H bending	[9]
1215	C–H ring bend and in-plane rocking	[6]
1210	C–H-C stretching	[5,8]
1227	N−N stretching mode	[7]
1250-1350	N-O symmetric stretching (Nitramine)	[7,8,10]
1270-1300	N-O symmetric stretching (Nitrate ester)	[8,10]
1315-1375	N-O symmetric stretching (Nitro-aromatic)	[5,6,8,10]
1487-1590	N-O asymmetric stretching (Nitro-aromatic)	[5,7,10]
1510	CH_2 scissoring	[8]
1500-1630	N-O asymmetric stretching (Nitramine)	[7,8,10,11]
1577	C–C stretching	[9]
1610-1660	N-O asymmetric stretching (Nitrate ester)	[8,10]
1615	C═C aromatic stretching	[6]
3073 - 2906	C−H stretching	[7,11]

13. SERS OF EXPLOSIVES: DATA FOR LOD STUDY

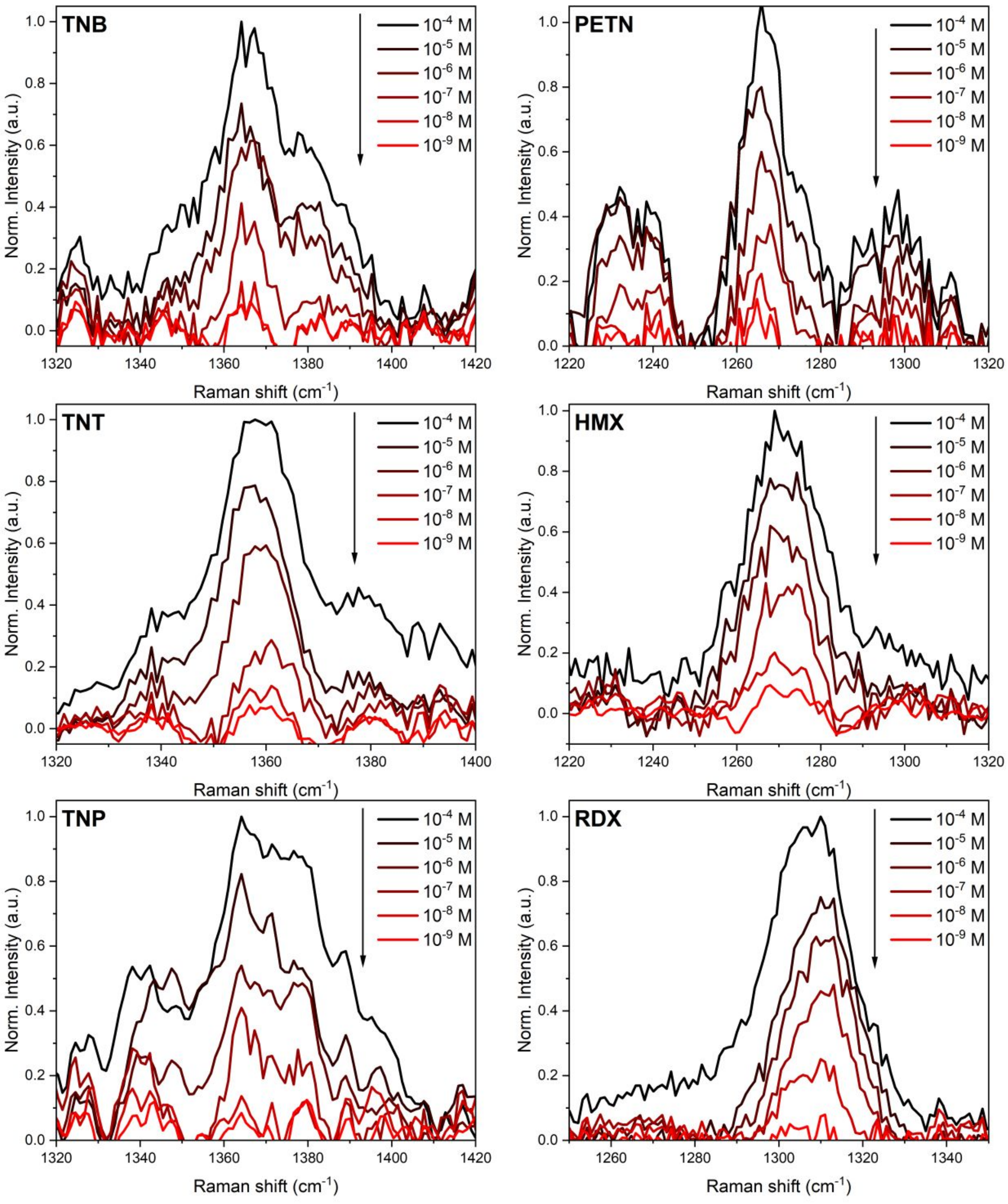

Figure S10: SERS spectra of $v_s(NO_2)$ peak for all explosives used in LoD

14. Relative standard deviation

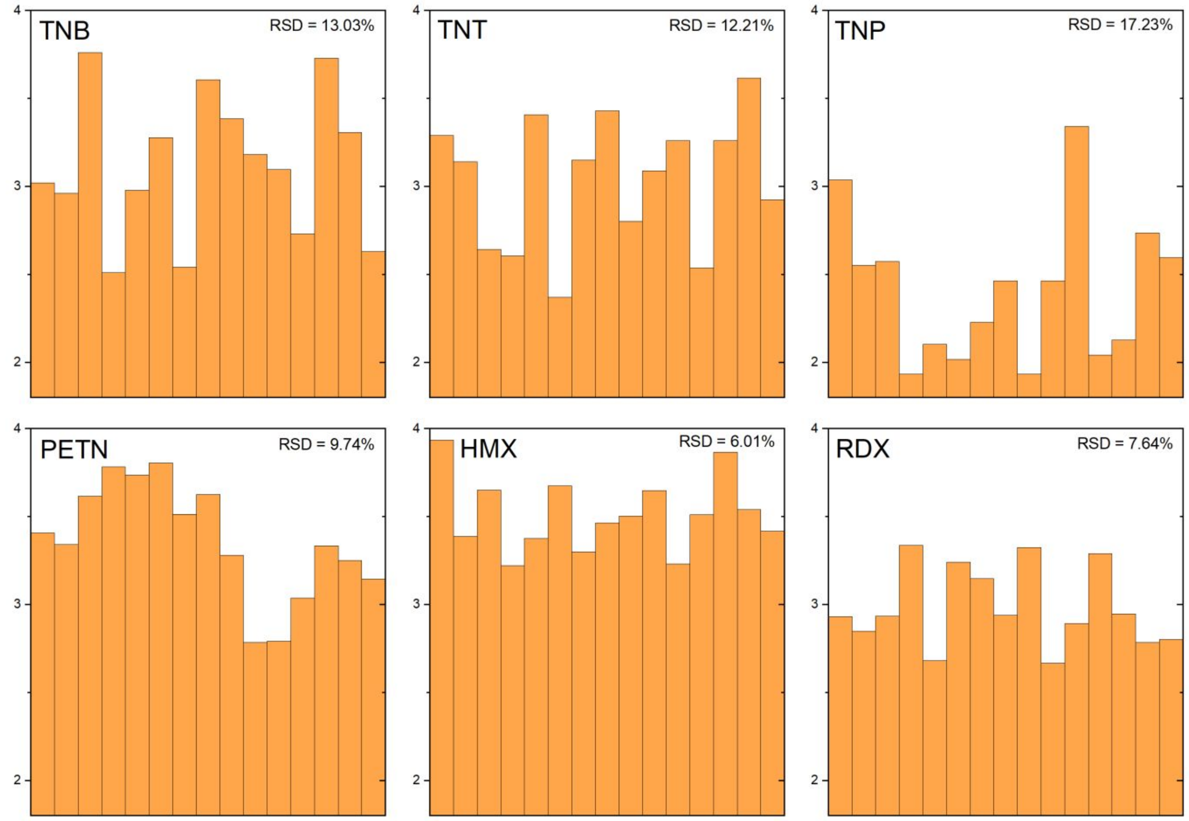

Figure S11: Standardised intensities of $v_s(NO_2)$ peak (range 1200-1400 cm^{-1}) for all explosives

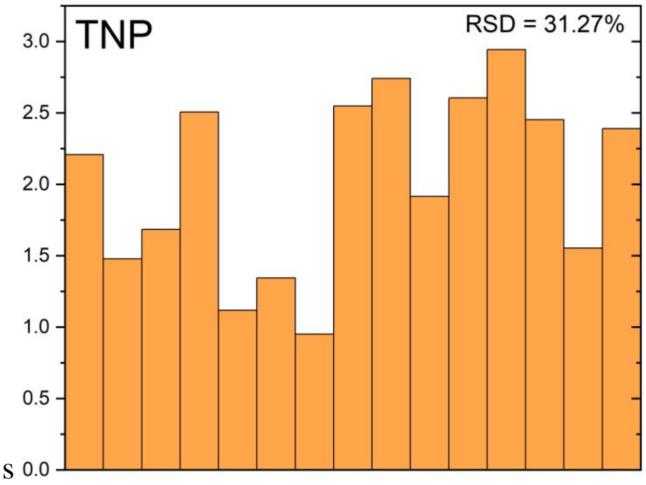

Figure S12: Standardised intensities of $v_{as}(NO_2)$ peak (range 1500-1600 cm^{-1}) for TNP

15. SERS VS UV-VIS LIMIT OF DETECTION

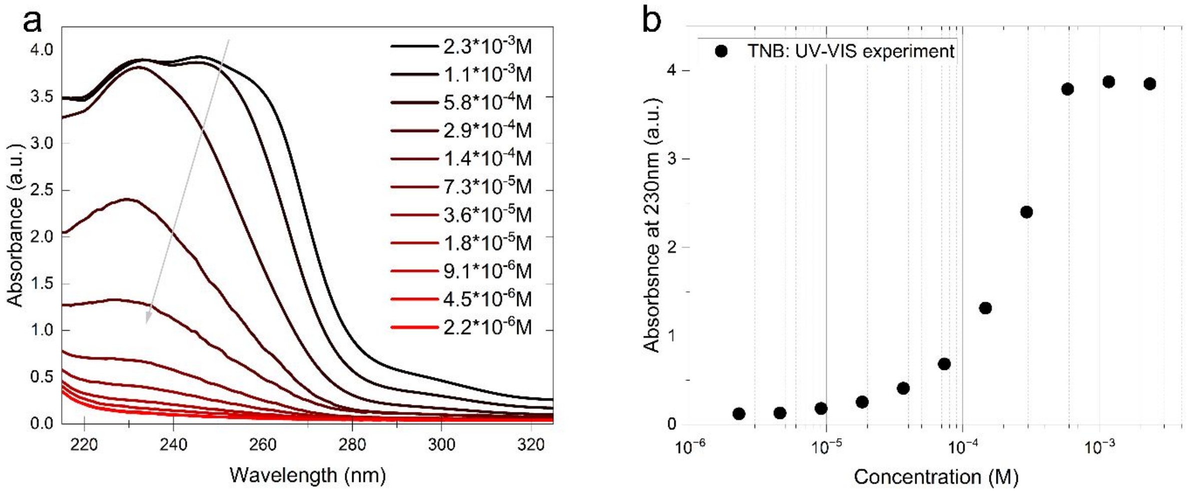

Figure S13: (a) UV-Vis absorption curves of the Aromatic TNB with varying concentrations on serial dilution ladder; (b) UV-Vis limit of detection

16.PCA+K-NN OPTIMIZATION

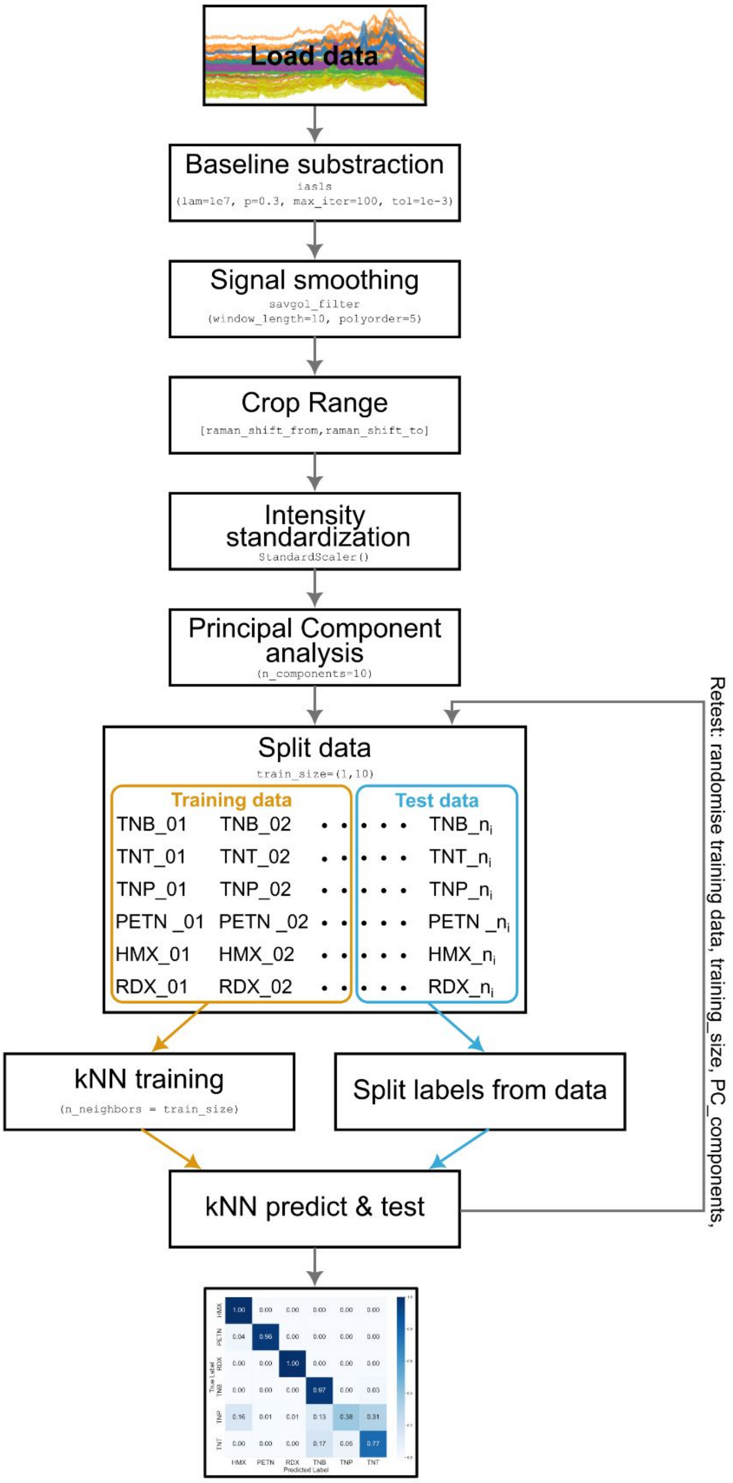

Figure S14: Schematic of kNN training and optimisation procedure

To accelerate the analysis and increase the credibility of the data, a multivariate statistical principal component analysis (PCA) was performed to reduce the dimensionality of the data. Prior to PCA, the raw data were subjected to baseline correction (Improved Asymmetric Least Squares method), followed by Savitzky-Golay filtering to smooth the profile shapes and

minimize the potential effects of small shifts in Raman peak positions, and finally the data was normalized to minimize the effects of scale on PCA.[12,13]

17. References

(1) Nikiforov, A. Y.; Leys, C.; Gonzalez, M. A.; Walsh, J. L. Electron Density Measurement in Atmospheric Pressure Plasma Jets: Stark Broadening of Hydrogenated and Non-Hydrogenated Lines. *Plasma Sources Sci Technol* 2015, *24* (3), 034001. https://doi.org/10.1088/0963-0252/24/3/034001.

(2) Bruggeman, P.; Schram, D.; González, M. Á.; Rego, R.; Kong, M. G.; Leys, C. Characterization of a Direct Dc-Excited Discharge in Water by Optical Emission Spectroscopy. *Plasma Sources Sci Technol* 2009, *18* (2), 025017. https://doi.org/10.1088/0963-0252/18/2/025017.

(3) Gigosos, M. A.; González, M. Á.; Cardeñoso, V. Computer Simulated Balmer-Alpha, -Beta and -Gamma Stark Line Profiles for Non-Equilibrium Plasmas Diagnostics. *Spectrochim Acta Part B At Spectrosc* 2003, *58* (8), 1489–1504. https://doi.org/10.1016/S0584-8547(03)00097-1.

(4) Sakiyama, Y.; Graves, D. B.; Chang, H. W.; Shimizu, T.; Morfill, G. E. Plasma Chemistry Model of Surface Microdischarge in Humid Air and Dynamics of Reactive Neutral Species. *J Phys D Appl Phys* 2012, *45* (42). https://doi.org/10.1088/0022-3727/45/42/425201.

(5) Dasary, S. S. R.; Singh, A. K.; Senapati, D.; Yu, H.; Ray, P. C. Gold Nanoparticle Based Label-Free SERS Probe for Ultrasensitive and Selective Detection of Trinitrotoluene. *J Am Chem Soc* 2009, *131* (38), 13806–13812. https://doi.org/10.1021/ja905134d.

(6) Jamil, A. K. M.; Izake, E. L.; Sivanesan, A.; Fredericks, P. M. Rapid Detection of TNT in Aqueous Media by Selective Label Free Surface Enhanced Raman Spectroscopy. *Talanta* 2015, *134*, 732–738. https://doi.org/10.1016/j.talanta.2014.12.022.

(7) Torres, P.; Mercado, L.; Cotte, I.; Hernández, S. P.; Mina, N.; Santana, A.; Chamberlain, R. T.; Lareau, R.; Castro, M. E. Vibrational Spectroscopy Study of β and α RDX Deposits. *J Phys Chem B* 2004, *108* (26), 8799–8805. https://doi.org/10.1021/jp0373550.

(8) Ghosh, M.; Wang, L.; Asher, S. A. Deep-Ultraviolet Resonance Raman Excitation Profiles of NH 4 NO 3 , PETN, TNT, HMX, and RDX. *Appl Spectrosc* 2012, *66* (9), 1013–1021. https://doi.org/10.1366/12-06626.

(9) Gao, W.; Wang, T.; Zhu, C.; Sha, P.; Dong, P.; Wu, X. A 'Sandwich' Structure for Highly Sensitive Detection of TNT Based on Surface-Enhanced Raman Scattering. *Talanta* 2022, *236*, 122824. https://doi.org/10.1016/j.talanta.2021.122824.

(10) Lewis, I. R.; Daniel, N. W.; Griffiths, P. R. Interpretation of Raman Spectra of Nitro-Containing Explosive Materials. Part I: Group Frequency and Structural Class Membership. *Appl Spectrosc* 1997, *51* (12), 1854–1867. https://doi.org/10.1366/0003702971939686.

(11) Dreger, Z. A.; Gupta, Y. M. High Pressure Raman Spectroscopy of Single Crystals of Hexahydro-1,3,5-Trinitro-1,3,5-Triazine (RDX). *J Phys Chem B* 2007, *111* (15), 3893–3903. https://doi.org/10.1021/jp0681092.

(12) Guo, S.; Popp, J.; Bocklitz, T. Chemometric Analysis in Raman Spectroscopy from Experimental Design to Machine Learning–Based Modeling. *Nat Protoc* 2021, *16* (12), 5426–5459. https://doi.org/10.1038/s41596-021-00620-3.

(13) He, S.; Zhang, W.; Liu, L.; Huang, Y.; He, J.; Xie, W.; Wu, P.; Du, C. Baseline Correction for Raman Spectra Using an Improved Asymmetric Least Squares Method. *Anal. Methods* 2014, *6* (12), 4402–4407. https://doi.org/10.1039/C4AY00068D.
